# Evaluation of Dimebon in cellular model of Huntington's disease

**DOI:** 10.1186/1750-1326-3-15

**Published:** 2008-10-21

**Authors:** Jun Wu, Qin Li, Ilya Bezprozvanny

**Affiliations:** 1 Department of Physiology, UT Southwestern Medical Center at Dallas, TX 75390, USA

## Abstract

**Background:**

Dimebon is an antihistamine compound with a long history of clinical use in Russia. Recently, Dimebon has been proposed to be useful for treating neurodegenerative disorders. It has demonstrated efficacy in phase II Alzheimer's disease (AD) and Huntington's disease (HD) clinical trials. The mechanisms responsible for the beneficial actions of Dimebon in AD and HD remain unclear. It has been suggested that Dimebon may act by blocking NMDA receptors or voltage-gated Ca^2+ ^channels and by preventing mitochondrial permeability pore transition.

**Results:**

We evaluated the effects of Dimebon in experiments with primary striatal neuronal cultures (MSN) from wild type (WT) mice and YAC128 HD transgenic mice. We found that Dimebon acts as an inhibitor of NMDA receptors (IC50 = 10 μM) and voltage-gated calcium channels (IC50 = 50 μM) in WT and YAC128 MSN. We further found that application of 50 μM Dimebon stabilized glutamate-induced Ca^2+ ^signals in YAC128 MSN and protected cultured YAC128 MSN from glutamate-induced apoptosis. Lower concentrations of Dimebon (5 μM and 10 μM) did not stabilize glutamate-induced Ca^2+ ^signals and did not exert neuroprotective effects in experiments with YAC128 MSN. Evaluation of Dimebon against a set of biochemical targets indicated that Dimebon inhibits α-Adrenergic receptors (α_1A_, α_1B_, α_1D_, and α_2A_), Histamine H_1 _and H_2 _receptors and Serotonin 5-HT_2c_, 5-HT_5A_, 5-HT_6 _receptors with high affinity. Dimebon also had significant effect on a number of additional receptors.

**Conclusion:**

Our results suggest that Ca^2+ ^and mitochondria stabilizing effects may, in part, be responsible for beneficial clinical effects of Dimebon. However, the high concentrations of Dimebon required to achieve Ca^2+ ^stabilizing and neuroprotective effects in our *in vitro *studies (50 μM) indicate that properties of Dimebon as cognitive enhancer are most likely due to potent inhibition of H1 histamine receptors. It is also possible that Dimebon acts on novel high affinity targets not present in cultured MSN preparation. Unbiased evaluation of Dimebon against a set of biochemical targets indicated that Dimebon efficiently inhibited a number of additional receptors. Potential interactions with these receptors need to be considered in interpretation of results obtained with Dimebon in clinical trials.

## Background

Huntington disease (HD) is an inherited, incurable, autosomal dominant disease caused by the expansion of CAG trinucleotide repeats in the first exon of the huntingtin gene [1,2]. It is characterized by progressive neurodegeneration resulting in motor abnormalities including chorea and psychiatric disturbance with gradual dementia. HD is fatal and causes death within 15–20 years of the onset of the symptoms. The mutant huntingtin (Htt) with expanded polyglutamine (polyQ) is widely expressed in the brain and peripheral tissues but causes selective and most prominent loss of medium spiny neurons in striatum (MSN) which leads to the major clinical abnormalities that characterize the disease. The exact cause of neuronal loss in HD remains unknown [3]. Recent evidence indicates that dysregulation of glutamate and Ca^2+ ^(calcium) signaling in MSN play an important role in HD pathogenesis [4]. The "Ca^2+ ^hypothesis of HD" suggests that Ca^2+ ^signaling inhibitors may have a therapeutic value for treatment of HD [4]. Abnormal neuronal Ca^2+ ^signaling has also been proposed to play an important role in Alzheimer's disease [5,6].

Dimebon is a drug that has been developed and used as an antihistamine in Russia since 1983. Recently, Dimebon has been proposed to be useful for treating neurodegenerative disorders [7]. Dimebon demonstrated significant positive effects in six-month randomized, double-blinded, placebo-controlled phase II trial of 183 patients with mild to moderate Alzheimer's disease (AD) conducted by Medivation [8]. The phase III trial of Dimebon in AD will soon be initiated. Dimebon also demonstrated efficacy in phase 2 trial of patients with Huntington's disease (HD) conducted by Medivation and Huntington Study Group (DIMOND). Despite extremely encouraging results in clinical trials, the mechanisms responsible for beneficial actions of Dimebon in AD and HD remain poorly understood. Previous reports suggested that Dimebon may act as an inhibitor of NMDA receptors [9], voltage-gated Ca^2+ ^channels [10] or as a blocker of mitochondrial permeability transition pore [11]. These potential targets indicated that Dimebon may act by stabilizing neuronal Ca^2+ ^signaling, which may explain clinical benefits observed in HD and AD trials. In the present study, we evaluated the ability of Dimebon to inhibit NMDA receptors and voltage-gated Ca^2+ ^channels in cultured MSN from wild type mice and from the YAC128 HD transgenic mice model. We also evaluated neuroprotective effects of Dimebon in previously developed glutamate-toxicity assay with cultured YAC128 MSN [12]. We concluded that Ca^2+ ^and the mitochondria stabilizing effects of Dimebon may only in part be responsible for beneficial effects in human clinical trials and additional mechanisms of Dimebon's actions need to be uncovered to explain its beneficial clinical actions in HD and AD trials.

## Methods

### Drugs

**Figure 1:**
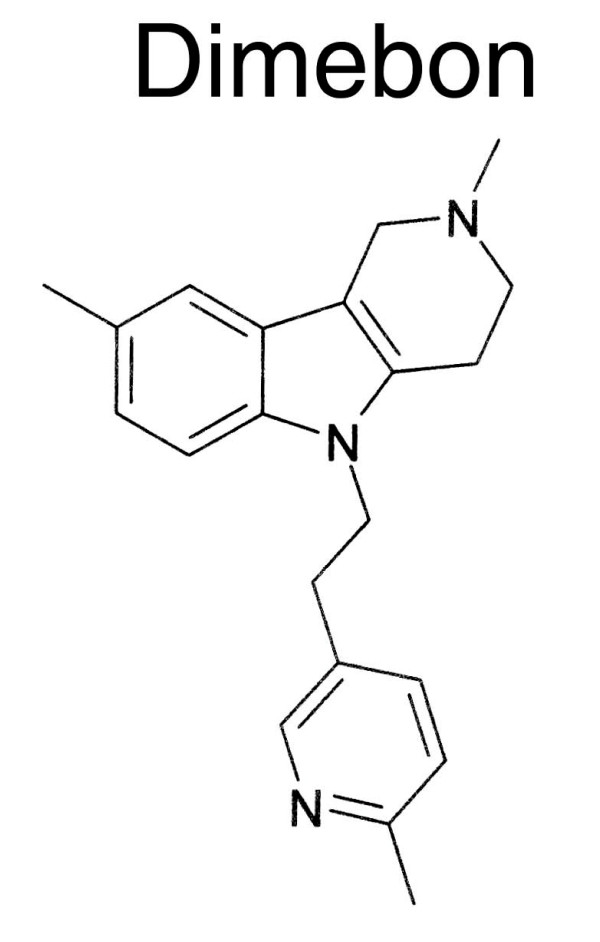
**Chemical structure of Dimebon**. Chemical structure of 2,3,4,5-Tetrahydro-2,8-dimethyl-5-(2-(6-methyl-3-pyridyl)ethyl)-1H-pyrido(4,3-b)indole (CAS 3613-73-8)

The Glutamate was from Tocris; and the Fura-2/AM was purchased from Sigma. Dimebon (2,3,4,5-Tetrahydro-2,8-dimethyl-5-(2-(6-methyl-3-pyridyl)ethyl)-1H-pyrido(4,3-b)indole, C_21_H_25_N_3_, CAS 3613-73-8) was synthesized by Nanosyn Inc. The synthesized material was 97.3% pure by HPLC-UV_275 _analysis. The structure of Dimebon (Fig 1) was independently confirmed by mass-spectroscopy and 1D and 2D NMR analysis (Bruker 500 NMR spectrometer). The proton and carbon 1D NMR spectra of the Dimebon sample used in our experiments are provided as Additional Files 1 and 2. In our experiments, Dimebon was dissolved in DMSO as concentrated stock (100 mM) and further diluted to its final concentration in Neurobasal-A medium for *in vitro *HD assay, in extracellular recording solutions for electrophysiology experiments or in artificial cerebrospinal fluid (ACSF) for Ca^2+ ^imaging experiments.

### Primary neuronal cultures

YAC128 mice (FVBN/NJ background strain) were obtained from Jackson Labs (stock number 004938). The male YAC128 mice were crossed to wild type (WT) female FVBN/NJ mice and P1-P2 pups were collected and genotyped by PCR. The primary cultures of striatal medium spiny neurons (MSN) were established from YAC128 and control wild type pups as we previously described [12-15]. Striata were dissected, diced and digested with trypsin. After dissociation, neurons were plated on poly-L-lysine (Sigma) coated 12 mm round coverslips (Assistent) in Neurobasal-A medium supplemented with 2% B27, 1 mM glutamine and penicillin-streptomycin (all from Invitrogen) and kept at 37°C in a 5% CO_2 _environment.

### Calcium imaging experiments

Ca^2+ ^imaging experiments with 13–14 DIV (days in *vitro*) MSN cultures were performed as previously described [12,14-16], using a DeltaRAM illuminator, an IC-300 camera, and IMAGEMASTER PRO software (all from PTI). The MSN were loaded with 5 μM fura-2 AM (Molecular Probes) for 45 min at 37°C in artificial cerebrospinal fluid (ACSF, containing the following: 140 mM NaCl, 5 mM KCl, 1 mM MgCl_2_, 2 mM CaCl_2_, 10 mM Hepes, pH7.3). Coverslips were mounted onto a recording/perfusion chamber (RC-26G, Warner Instruments) and positioned on the movable stage of an Olympus (Melville) IX-70 inverted microscope. The cells were maintained in ACSF at 37°C during experiments (PH1 heater, Warner Instruments). Images at 340 and 380 nm excitation wavelengths were acquired every 6 s and shown as 340/380 image ratios. Baseline (1–3 min) measurements were obtained before first pulse of glutamate. The 20 μM glutamate solution was dissolved in ACSF and 1-min pulses of 37°C glutamate solution (SH-27B in-line solution heater, Warner Instruments) were applied by using a valve controller (VC-6, Warner Instruments) driven by a square-pulse electrical wave-form generator (Model 148A, Wavetek). 10 μM or 50 μM Dimebon was dissolved in ACSF or 20 μM glutamate solution for the Dimebon application.

### Electrophysiology for NMDAR and voltage-gated Ca^2+ ^channels

Whole-cell patch-clamp recordings of NMDAR activity were performed with cultured MSN from WT and YAC128 mice at DIV9-10 as we previously described [15]. Medium spiny neurons were distinguished based on morphological identification and membrane capacitance ranging from 4–10 pF. A multi-barrel perfusion system was employed to achieve a rapid exchange of extracellular solutions as we previously described [15]. All drugs were prepared according to the specifications of the manufacturers and applied with a gravity-fed "sewer pipe" capillary array. Whole-cell currents were recorded using Axopatch 200B amplifiers (Axon Instruments). Data were filtered at 2 kHz and digitized at 5 Hz using a Digidata 1200 DAC unit (Axon Instruments). The online acquisition was done using pCLAMP software (Version 8, Axon Instruments). Cultured MSN were held at V_H _= -60 mV membrane potential. Standard extracellular solutions contained 140 mM NaCl, 5 mM KCl, 2.0 mM CaCl_2_, 10 mM HEPES (pH 7.4; 310 mOsm). The pipette solution contained 135 mM CsMeSO4, 10 mM HEPES, 5 mM 1,2-bis(2-aminophenoxy)ethane-N,N,N',N'-tetraacetic acid, 3 mM MgATP, 1 mM MgCl_2_, 0.3 mM GTP-tris. In all experiments, 50 μM glycine was added to both control and NMDA-containing extracellular solutions. 10 μM CNQX and 0.1 μM TTX were added to the extracellular recording solution immediately before each experiment to block AMPA/kainate-type glutamate receptors and voltage-gated sodium currents, respectively.

Whole-cell patch-clamp recordings of voltage gated Ca^2+ ^currents in cultured MSN at DIV9 were performed according to the published procedures [17]. The recording chamber was perfused with extracellular solution containing 140 mM TEA-Cl, 10 mM BaCl_2_, 10 mM HEPES, 20 mM glucose, 0.001 mM tetrodotoxin (TTX), pH 7.4, with TEA-OH, 310 mOsm. The pipette solution contained 110 mM CsCl, 10 mM EGTA, 4 mM ATP-Mg, 0.3 mM GTP-Na, 25 mM HEPES, 10 mM Tris-phosphocreatine, 20 units/ml creatine phosphokinase, pH 7.3, with CsOH, 290 mOsm. Ba^2+ ^current traces were corrected for linear capacitive leak with online P/6 trace subtraction. MSN were voltage clamped at -80 mV, and test pulses were applied at 10 s intervals.

### *In vitro *HD assay

The *in vitro *HD assay with wild type and YAC128 MSN cultures was conduced as previously described [12,13]. Dimebon was added to the 14 DIV MSN at the final concentration of 5 μM, 10 μM or 50 μM. After 30 minutes incubation with Dimebon, the MSN were exposed for 7 h to 250 μM glutamate in Neurobasal-A added to the culture medium. During exposure to glutamate, the cells were maintained in a cell culture incubator (humidified 5% CO_2_, 37°C). Immediately after the treatment with glutamate, neurons were fixed for 30 min in 4% paraformaldehyde plus 4% sucrose in PBS (pH7.4), permeabilized for 5 min in 0.25% Triton-X-100, and stained by using the DeadEnd fluorometric TUNEL System (Promega). Nuclei were counterstained with 5 μM propidium iodine (PI) (Molecular Probes). Coverslips were extensively washed with PBS and mounted in Mowiol 4–88 (Polysciences). For quantification, six to eight randomly chosen microscopic fields containing 100–300 MSN each were cell-counted for YAC128 and wild type cultures. The number of TUNEL-positive neuronal nuclei was calculated as a fraction of PI-positive neuronal nuclei in each microscopic field. The fractions of TUNEL-positive nuclei determined for each microscopic field were averaged and the results are presented as means ± SE (n = number of fields counted).

### Evaluation against biochemical targets

The activity of Dimebon against selected set of biochemical targets was performed by MDS Pharma Services . The Dimebon was provided to MDS Pharma as dry powder, dissolved in DMSO and tested in 10 μM concentrations. The standard Lead Profiling + CYP450 screen was performed according to MDS Pharma specifications with additional custom-chosen targets added to the screen as indicated.

### Statistical analysis

All experiments were repeated at least three times. Data were evaluated for statistical significance by analysis using SigmaPlot t-test or One-Way ANOVA. Statistical difference was considered to be significant if p < 0.05.

## Results

### Dimebon inhibits glutamate-induced Ca^2+ ^increase in YAC128 MSN

**Figure 2:**
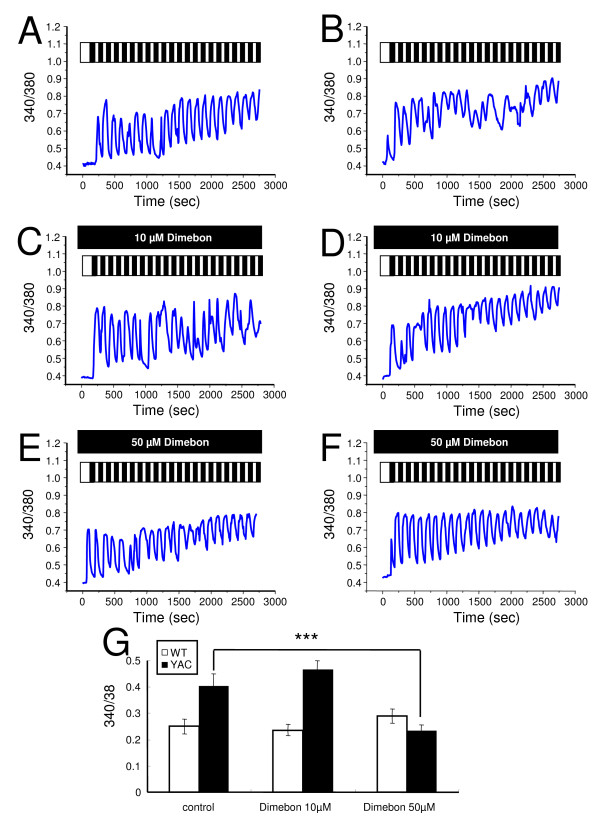
**Effects of Dimebon on glutamate-induced Ca^2+^signals**. (A-B), Repetitive application of 20 μM glutamate induces Ca^2+ ^signals in MSN from the WT (A) and YAC128 (B) mice. (C-D), The same experiment as in (A) and (B) was performed in the presence of 10 μM Dimebon with WT (C) and YAC128 (D) MSN. (E-F), The same experiment as in (A) and (B) was performed in the presence of 50 μM Dimebon with WT (E) and YAC128 (F) MSN. The traces shown on panels (A-F) are average traces from all MSN for each experimental group. (G) The average increase of basal Ca^2+ ^level (mean ± SE, n is the number of MSN analyzed) after 20 pulses of glutamate are shown for WT MSN (n = 16), YAC128 MSN (n = 21), WT MSN in the presence of 10 μM Dimebon (n = 44), YAC128 MSN in the presence of 10 μM Dimebon (n = 41), WT MSN in the presence of 50 μM Dimebon (n = 17) and YAC128 MSN in the presence of 50 μM Dimebon (n = 44).

To test the postulated "Ca^2+ ^stabilizing" effects of Dimebon, in the first series of experiments, we compared Ca^2+ ^responses induced by glutamate application to wild type (WT) and YAC128 MSN at 13–14 DIV. In our previous studies, we found that repetitive pulses of 20 μM glutamate resulted in a bigger elevation of cytosolic Ca^2+ ^levels in the YAC128 MSN compared with that in WT MSN [12,14,15]. To test effects of Dimebon on glutamate-induced Ca^2+ ^signals, we applied 20 pulses of 20 μM glutamate (each pulse 1 min in duration, followed by a 1 min washout) in the presence of 10 μM or 50 μM Dimebon. Control experiments were performed in the absence of Dimebon. The intracellular Ca^2+ ^concentration in the experiments was continuously monitored by Fura-2 imaging and the 340/380 ratio was used to quantitatively determine the concentration of the intracellular Ca^2+ ^([Ca^2+^]_i_). The increase in Ca^2+ ^was calculated as a difference between basal values of Ca^2+ ^prior to glutamate application and at completion of "20 glutamate pulses" protocol in the same cell. On average, the increase in Ca^2+ ^was 0.250 ± 0.029 for WT MSN and 0.403 ± 0.046 for YAC128 MSN (Figs 2A, B, G). Thus, in agreement with our previous findings [12,14,15], the increase in Ca^2+ ^was significantly higher in YAC128 MSN than in WT MSN. In the presence of 10 μM Dimebon, the increase in Ca^2+ ^was 0.236 ± 0.021 for WT MSN and 0.461 ± 0.034 for YAC128 MSN (Figs 2C, D, G). Thus, incubation with 10 μM Dimebon had no significant effect on the glutamate-induced Ca^2+ ^increase in WT or YAC128 MSN. In the presence of 50 μM Dimebon, the increase in Ca^2+ ^was 0.290 ± 0.027 for WT MSN and 0.234 ± 0.022 for YAC128 MSN (Figs 2E, F, G). Thus, 50 μM Dimebon significantly reduced the glutamate-induced Ca^2+ ^increase of YAC128 MSN without affecting the Ca^2+ ^signals in WT MSN. These results indicate that Dimebon exerts "Ca^2+ ^stabilizing" effects in YAC128 MSN at 50 μM but not at 10 μM concentration.

### NMDAR inhibition by Dimebon

**Figure 3:**
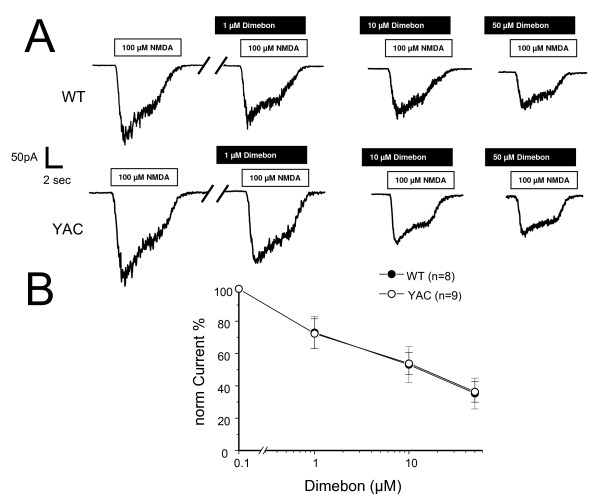
**Effects of Dimebon on NMDA-evoked currents**. (*A*) Representative traces of NMDA-evoked current recorded from DIV9-10 WT and YAC128 MSN. The white bar represents application of 100 μM NMDA in the presence of 50 μM glycine and black bar represents application of 1 μM, 10 μM and 50 μM Dimebon as indicated. (*B*) Dose-dependence of Dimebon block. The peak NMDA-induced currents were normalized to the peak NMDA-induced currents in the same cell in the absence of Dimebon. The normalized data at each Dimebon concentration were averaged from several experiments and shown as mean ± SE (n = 8 for WT and n = 9 for YAC128 MSN). The IC50 = 10 μM for WT and YAC128 MSN.

Previous reports suggested that Dimebon may act as an inhibitor of NMDA receptors [9]. In the next series of experiments, we evaluated the ability of Dimebon to block NMDA-activated currents in DIV9-10 WT and YAC128 MSN cultures. The MSN were voltage-clamped at -60 mV and the currents were evoked by local and rapid application of 100 μM NMDA using multi-barrel perfusion system ("sewer pipe"). Consistent with previous findings [15], we found that NMDA induced much larger currents in YAC128 MSNs than in the WT littermate cultures (Fig 3A). Application of 1 μM, 10 μM, or 50 μM of Dimebon caused a significant reduction in the size of NMDA-induced currents in both WT and YAC128 MSN (Fig 3A). The inhibitory effects of Dimebon were reversible and the size of NMDA currents quickly recovered following a washout of Dimebon (data not shown). To compare results from different experiments, we normalized the peak amplitude of NMDA-evoked currents to the amplitude recorded in the absence of Dimebon in the same cell and averaged normalized data from multiple experiments (from at least 4 independent cultures established from YAC128 and WT). Analysis of obtained results suggested that Dimebon blocks NMDAR-currents in WT and YAC128 MSN with IC50 = 10 μM (Fig 3B). The YAC128 MSN have increased contribution of NR2B subtype of NMDAR when compared to WT MSN [15,18]. Our results suggest that Dimebon does not display selectivity for NR2B subtype of NMDAR, as both WT and YAC128 MSN currents were inhibited with similar potency (Fig 3B). In general, our results are consistent with previous studies of Dimebon's inhibitory effects on NMDAR [9].

### Voltage-gated calcium channel inhibition by Dimebon

**Figure 4:**
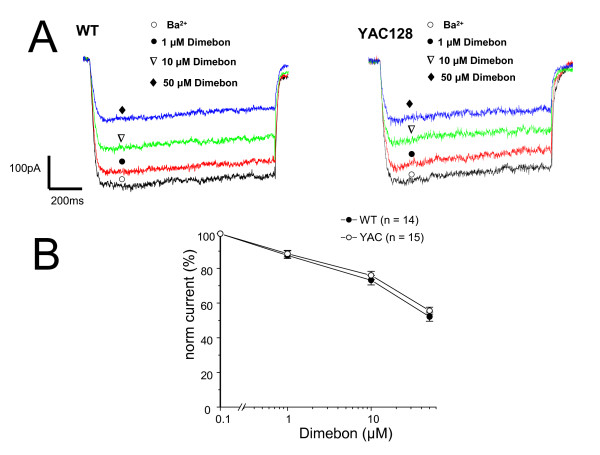
**Effects of Dimebon on voltage-gated calcium currents**. (A) Representative traces of voltage-gated Ca^2+ ^currents evoked by membrane depolarization from -80 mV holding potential to 0 mV in DIV9 WT and YAC128 MSN. The current waveforms recorded in the same cell in the absence of Dimebon (open circle), and in the presence of 1 μM (filled circle), 10 μM (open triangle) and 50 μM (filled diamond) of Dimebon are shown. (*B*) Dose-dependence of Dimebon block of voltage-gated Ca^2+ ^channels. The peak voltage-gated Ca^2+ ^currents were normalized to the peak voltage-gated Ca^2+ ^currents recorded in the same cell in the absence of Dimebon. The normalized data at each Dimebon concentration were averaged from several experiments and shown as mean ± SE (n = 14 for WT and n = 15 forYAC128 MSN). The IC50 = 50 μM for WT and YAC128 MSN.

High voltage-activated Ca^2+ ^channels have been proposed to be another target of Dimebon [10]. In the next series of experiments, we evaluated effects of Dimebon on high voltage-activated Ca^2+ ^currents recorded in wild type (WT) and YAC128 MSN cultures at DIV9-10. Whole-cell patch-clamp recordings of currents in cultured MSN were performed according to the published procedures [17] using 10 mM Ba^2+ ^as a current carrier. The MSN were voltage clamped at -80 mV, and test pulses to 0 mV were applied at 10 s intervals. Consecutive application of 1 μM, 10 μM, and 50 μM of Dimebon resulted in a progressive reduction in the size of depolarization-evoked currents (Fig 4A). To compare results from different experiments, we normalized the peak size of voltage-gated Ca^2+ ^currents to the amplitude recorded in the absence of Dimebon in the same cell and averaged normalized data from multiple experiments (from at least 4 independent cultures established from YAC128 and WT). Analysis of obtained results suggested that Dimebon blocks high-voltage activated Ca^2+ ^channels in WT and YAC128 MSN with IC50 = 50 μM (Fig 4B). Dimebon demonstrated similar potency in WT and YAC128 MSN cultures (Fig 4B). In general, our results are consistent with previous studies of Dimebon's inhibitory effects on high voltage-activated Ca^2+ ^channels [10].

### Neuroprotective effects of Dimebon in *in vitro *HD assay

**Figure 5:**
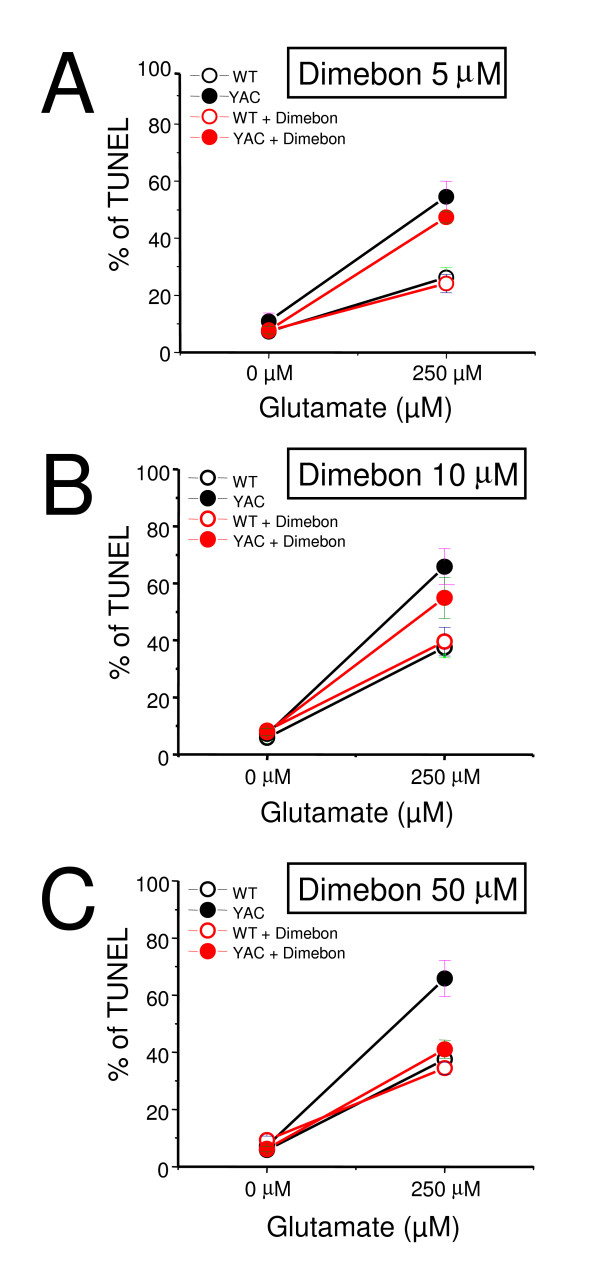
**Evaluation of Dimebon in *in vitro *HD assay**. Glutamate-induced apoptosis of WT and YAC128 MSN treated with Dimebon at different concentrations. WT and YAC128 MSN at 14 DIV were exposed to 250 μM glutamate for 7 h, fixed, permeabilized and analyzed by TUNEL staining and PI counterstaining. The Dimebon was added 30 min before the application of glutamate. The fraction of TUNEL-positive is plotted against glutamate concentration for WT (open circle) and YAC128 (YAC, filled circles) mice. The data are shown as mean ± SE (n = 6–8 microscopic fields, 100–300 MSN per field). The results in the absence (black symbols) and presence (red symbols) obtained in the presence of 5 μM of Dimebon (A) 10 μM Dimebon (B) and 50 μM Dimebon (C) are compared.

To evaluate neuroprotective effects of Dimebon, we performed a series of glutamate toxicity experiments with WT and YAC128 MSN DIV14 cultures. In the absence of glutamate approximately 5–10% of neurons are apoptotic in both wild type (WT) and YAC128 MSN cultures. Following exposure to 250 μM glutamate, the fraction of apoptotic WT MSN is increased to 25–40% and the fraction of apoptotic YAC128 MSN is increased to 55–70% (Table 1). The difference between the glutamate-induced apoptosis of YAC128 and WT MSN is highly significant and constitutes a quantitative basis for the *in vitro *HD assay we have previously described [12,13,15]. The neuroprotective effects of Dimebon were evaluated at 5 μM, 10 μM and 50 μM concentrations using the *in vitro *HD assay (Table 1, Fig 5). In these experiments, Dimebon was added 30 minutes prior to the exposure of MSN cultures to glutamate. We found that 5 μM and 10 μM Dimebon had no significant effects on the glutamate-induced apoptosis of YAC128 MSN (Table 1, Figs 5A, B). At 50 μM concentration, Dimebon showed significant protective effects in the *in vitro *HD assay (Table 1, Fig 5C).

**Table 1:** Effects of Dimebon on glutamate-induced apoptosis in WT and YAC128 MSN.

	**WT (% TUNEL-positive)**				**YAC128 (% TUNEL-positive)**			
	
**Drug treatment**	**0 μM glutamate**		**250 μM glutamate**		**0 μM glutamate**		**250 μM glutamate**	
**Dimebon**								
5 μM	7.53 ± 2.15	(7.25 ± 1.11)^a^	24.14 ± 3.22	(26.26 ± 3.50)	7.78 ± 1.29	(10.93 ± 2.89)	47.35 ± 1.95	(54.53 ± 5.40)
10 μM	8.34 ± 1.40	(5.88 ± 0.62)	39.58 ± 5.01	(37.59 ± 3.71)	7.86 ± 1.21	(7.24 ± 0.92)	54.90 ± 7.17	(65.84 ± 6.27)
50 μM	7.14 ± 1.19	(5.88 ± 0.62)	34.50 ± 2.12	(37.59 ± 3.71)	6.19 ± 0.96	(7.24 ± 0.92)	41.08 ± 3.25*	(65.84 ± 6.27)

The fraction of apoptotic (TUNEL-positive) neurons is shown for wild type (WT) and YAC128 MSN without glutamate application and following application of 250 μM glutamate for 7 h. The experiments were conduced in the presence of 5, 10 and 50 μM Dimebon as indicated.

^a^The number in parenthesis is the untreated control cells in the same batch of MSN culture.

*P < 0.05, compared with control cells and show protective effect.

### Evaluation of Dimebon against a set of biochemical targets

**Figure 6:**
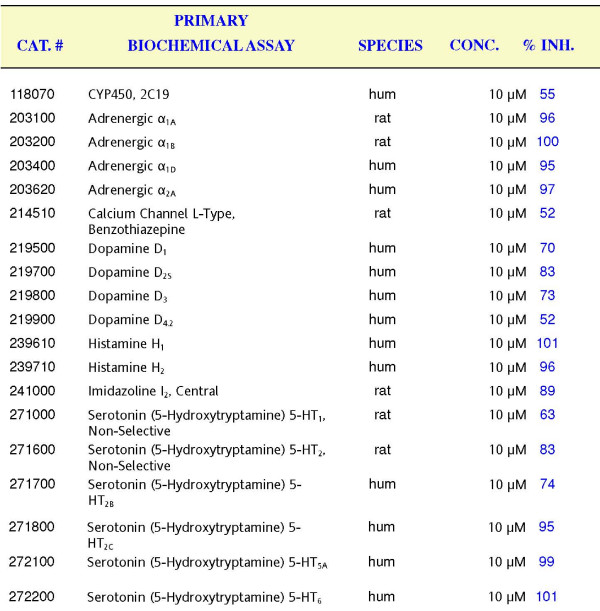
**Significant biochemical targets of Dimebon**. Significant biochemical targets of Dimebon are shown. The Cat numbers refer to the MDS Pharma Services assay specification. The receptors and species (human or rat) are listed. Dimebon was tested at 10 μM concentration. The % inhibition is shown for each receptor. Significant targets are defined as % inhibition > 50%.

To further understand potential mechanisms responsible for clinical effects of Dimebon, we evaluated Dimebon against a set of selected biochemical targets. Dimebon evaluation was performed by MDS Pharma Services . The standard Lead Profiling + CYP450 screen was performed with additional custom-selected targets added to the screen. The results of the screen obtained at 10 μM of Dimebon are provided as Additional File 3. The significant effect (> 50% inhibition) by 10 μM of Dimebon in binding or biochemical experiments was observed for a set of receptors listed on Fig 6. It is apparent that Dimebon very efficiently inhibits α-Adrenergic receptors (α_1A_, α_1B_, α_1D_, and α_2A_), Histamine H_1 _and H_2 _receptors and Serotonin 5-HT_2c_, 5-HT_5A_, 5-HT_6_receptors (Fig 6). For all of these receptors 10 μM of Dimebon resulted in > 90% inhibition. Dimebon also significantly inhibited Dopamine D_1_, D_2S_, D_3 _receptors, Imidazoline I_2 _receptor, Serotonin 5-HT_2 _and 5-HT_2B _receptors (70–80% inhibition). Low affinity (50–60% inhibition) interactions were also observed for CYP450, 2C19 receptor, voltage-gated L-type Ca^2+ ^channel, Dopamine D_4.2 _receptor, and Serotonin 5-HT_1 _receptor. Overall, these results suggested broad pharamacological profile of Dimebon.

## Discussion

Dimebon demonstrated significant positive effects in phase II AD clinical trial conducted by Medivation in Russia [8]. It has also demonstrated efficacy in a phase 2 trial of patients with Huntington's disease (HD) conducted by Medivation and Huntington Study Group (DIMOND). Despite extremely encouraging results in clinical trials, the mechanisms responsible for the beneficial actions of Dimebon in AD and HD remain poorly understood. Here, we evaluated neuroprotective effects of Dimebon in a previously developed cellular model of HD [12]. We also tested the ability of Dimebon to function as a "Ca^2+ ^signaling stabilizer" and an inhibitor of NMDAR and voltage-gated Ca^2+ ^channels in Ca^2+ ^imaging and electrophysiological experiments with wild type and YAC128 MSN cultures. Consistent with the previous reports [9,10], we found that Dimebon indeed inhibits NMDAR and voltage-gated Ca^2+ ^channels. The IC50 for inhibitory actions of Dimebon was equal to 10 μM for NMDAR and 50 μM for voltage-gated Ca^2+ ^channels in our experiments (Figs 3B and 4B). At 50 μM concentration, Dimebon also exerted Ca^2+ ^stabilizing and neuroprotective effects in YAC128 MSN preparation (Figs 2G and 5C, Table 1). At the concentration of 10 μM, Dimebon was not effective in these *in vitro *assays (Figs 2G and 5B, Table 1). Similar efficacy of Dimebon was observed in experiments with the *Drosophila *model of HD [19]. Consistent with our findings, neuroprotective effects of Dimebon in the *Drosophila *model of HD were observed in the 50 – 100 μM concentration range [19]. The protective effects of Dimebon in the *in vitro *HD assay are in quantitative agreement with the "Ca^2+ ^stabilizing" effects of Dimebon (Fig 2G). Thus, neuroprotective effects of Dimebon observed in our experiments (Table 1, Fig 5C) most likely can be explained by "Ca^2+ ^stabilizing" effects of Dimebon resulting from inhibition of NMDAR (Fig 3) and voltage-gated Ca^2+ ^channels (Fig 4). It is also possible that Dimebon exerts additional beneficial actions at the level of neuronal mitochondria [11]. The concentration of Dimebon required to inhibit mitochondrial permeability pore transition in isolated mitochondria was in the range of 50 μM [11], the same concentration range as the neuroprotective effects observed in our experiments with YAC128 MSN cultures (Fig 5C, Table 1).

Using an identical experimental approach, in previous studies we demonstrated that clinically relevant NMDAR antagonist memantine (Namenda) was protective in the YAC128 MSN glutamate toxicity assay at 10 μM concentration [13]. Thus, Dimebon is 5-fold less effective than memantine when tested in the *in vitro *HD model. We also previously demonstrated that clinically relevant putative mitochondrial permeability pore inhibitors Nortriptyline, Desipramine, Trifluoperazine, and Maprotiline [20] were also protective in YAC128 MSN toxicity assay at 2 μM concentration [12]. The Dimebon was 25-fold less effective than these compounds when tested in the *in vitro *HD model.

Unbiased evaluation of Dimebon against a set of biochemical targets indicated that Dimebon efficiently inhibits α-Adrenergic receptors (α_1A_, α_1B_, α_1D_, and α_2A_), Histamine H_1 _and H_2 _receptors and Serotonin 5-HT_2c_, 5-HT_5A_, 5-HT_6 _receptors with high affinity (Fig 6). Dimebon also had significant effect on Dopamine D_1_, D_2S_, D_3 _receptors, Imidazoline I_2 _receptor, Serotonin 5-HT_2 _and 5-HT_2B _receptors (Fig 6). Interactions with these receptors need to be taken into consideration in interpretation of results obtained with Dimebon in HD and AD clinical trials.

## Conclusion

From our results, we concluded that the "Ca^2+ ^stabilizing" effects of Dimebon may, in part, be responsible for the clinical benefits observed in HD and AD trials. However, 50 μM concentration of Dimebon that is required to achieve "Ca^2+ ^stabilizing" and neuroprotective effects in our experiments is not likely to be achieved in human trials. In AD trials of Dimebon, the patients received 20 mg pills [8], which should not lead to concentrations higher than 0.6 μM assuming an ideal absorption and blood-brain-barrier brain permeability profile. Thus, most beneficial effects of Dimebon are likely to be due to its properties as a cognitive enhancer based on its ability to inhibit H1 histamine receptors with IC50 = 3.4 nM [7]. It is also possible that Dimebon acts on novel high affinity targets not present in cultured MSN preparation. Unbiased evaluation of Dimebon against a set of biochemical targets indicated that Dimebon efficiently inhibits α-Adrenergic receptors (α_1A_, α_1B_, α_1D_, and α_2A_), Histamine H_1 _and H_2 _receptors and Serotonin 5-HT_2c_, 5-HT_5A_, 5-HT_6 _receptors with high affinity. Dimebon also had significant effect on Dopamine D_1_, D_2S_, D_3 _receptors, Imidazoline I_2 _receptor, Serotonin 5-HT_2 _and 5-HT_2B _receptors. Potential interactions with these receptors need to be taken into consideration in interpretation of results obtained with Dimebon in HD and AD clinical trials. Further evaluation of Dimebon in AD and HD whole animal models will be required in order to better understand its mechanism of action.

## Competing interests

The authors declare that they have no competing interests.

## Authors' contributions

JW performed Ca^2+^ imaging and TUNEL experiments, prepared results for publication and drafted the manuscript. QL performed electrophysiological experiments and prepared results for publication. IB conceived the study, participated in its design and coordination and prepared the final version of the manuscript. All authors read and approved the final manuscript.

## Supplementary Material

Additional file 1 The proton 1D NMR spectra of Dimebon sample used in our experiments. The proton 1D NMR spectra of Dimebon sample is shown. Click here for file

Additional file 2 The carbon 1D NMR spectra of Dimebon sample used in our experiments. The carbon 1D NMR spectra of Dimebon sample is shown. Click here for file

Additional file 3 The results of Dimebon screening against a selected set of biochemical targets. The complete results obtained by MDS Pharma with a selected list of biochemical targets using 10 μM of Dimebon as a probe. The Cat numbers refer to the MDS Pharma Services assay specification. The receptors and species are listed. Dimebon was tested at 10 μM concentratio in duplicate. The % inhibition by Dimebon is shown numerically and graphically for each receptor. Significant targets (defined as % inhibition > 50%) are highlighted in yellow. Click here for file

## References

[B1] Vonsattel JP, DiFiglia M (1998). Huntington disease. J Neuropathol Exp Neurol.

[B2] The Huntington's Disease Collaborative Research and Group (1993). A novel gene containing a trinucleotide repeat that is expanded and unstable on Huntington's disease chromosomes. Cell.

[B3] Tobin AJ, Signer ER (2000). Huntington's disease: the challenge for cell biologists. Trends Cell Biol.

[B4] Bezprozvanny I, Hayden MR (2004). Deranged neuronal calcium signaling and Huntington disease. Biochem Biophys Res Commun.

[B5] Khachaturian ZS (1989). Calcium, membranes, aging, and Alzheimer's disease. Introduction and overview. Ann N Y Acad Sci.

[B6] Bezprozvanny I, Mattson MP (2008). Neuronal calcium mishandling and the pathogenesis of Alzheimer's disease. Trends Neurosci.

[B7] Bachurin S (2001). Antihistamine agent Dimebon as a novel neuroprotector and a cognition enhancer. Ann N Y Acad Sci.

[B8] Doody RS, Gavrilova SI, Sano M, Thomas RG, Aisen PS, Bachurin SO, Seely L, Hung D (2008). Effect of dimebon on cognition, activities of daily living, behaviour, and global function in patients with mild-to-moderate Alzheimer's disease: a randomised, double-blind, placebo-controlled study. Lancet.

[B9] Grigorev VV, Dranyi OA, Bachurin SO (2003). Comparative study of action mechanisms of dimebon and memantine on AMPA- and NMDA-subtypes glutamate receptors in rat cerebral neurons. Bull Exp Biol Med.

[B10] Lermontova NN, Redkozubov AE, Shevtsova EF, Serkova TP, Kireeva EG, Bachurin SO (2001). Dimebon and tacrine inhibit neurotoxic action of beta-amyloid in culture and block L-type Ca(2+) channels. Bull Exp Biol Med.

[B11] Bachurin SO, Shevtsova EP, Kireeva EG, Oxenkrug GF, Sablin SO (2003). Mitochondria as a target for neurotoxins and neuroprotective agents. Ann N Y Acad Sci.

[B12] Tang T-S (2005). Disturbed Ca2+ signaling and apoptosis of medium spiny neurons in Huntington's disease. Proc Natl Acad Sci USA.

[B13] Wu J, Tang T-S, Bezprozvanny I (2006). Evaluation of clinically-relevant glutamate pathway inhibitors in in vitro model of Huntington's disease. Neurosci Lett.

[B14] Tang TS, Chen X, Liu J, Bezprozvanny I (2007). Dopaminergic signaling and striatal neurodegeneration in Huntington's disease. J Neurosci.

[B15] Zhang H, Li Q, Graham RK, Slow E, Hayden MR, Bezprozvanny I (2008). Full length mutant huntingtin is required for altered Ca2+ signaling and apoptosis of striatal neurons in the YAC mouse model of Huntington's disease. Neurobiol Dis.

[B16] Tang T-S, Tu H, Chan EY, Maximov A, Wang Z, Wellington CL, Hayden MR, Bezprozvanny I (2003). Huntingtin and huntingtin-associated protein 1 influence neuronal calcium signaling mediated by inositol-(1,4,5) triphosphate receptor type 1. Neuron.

[B17] Bargas J, Howe A, Eberwine J, Cao Y, Surmeier DJ (1994). Cellular and molecular characterization of Ca2+ currents in acutely isolated, adult rat neostriatal neurons. J Neurosci.

[B18] Milnerwood AJ, Raymond LA (2007). Corticostriatal synaptic function in mouse models of Huntington's disease: early effects of huntingtin repeat length and protein load. J Physiol.

[B19] Hung D (2007). HYDROGENATED PYRIDO-INDOLE COMPOUNDS FOR THE TREATMENT OF HUNTINGTON'S DISEASE. WO/2007/041697, USA.

[B20] Stavrovskaya IG (2004). Clinically approved heterocyclics act on a mitochondrial target and reduce stroke-induced pathology. J Exp Med.

