# Supplementary material for: Evaluation of Dimebon in cellular model of Huntington's disease
Source: Mol Neurodegener. 2008 Oct 21;3:15. doi: 10.1186/1750-1326-3-15 (PMC2577671; doi:10.1186/1750-1326-3-15)
Supplement: Additional file 1 — The proton 1D NMR spectra of Dimebon sample used in our experiments. The proton 1D NMR spectra of Dimebon sample is shown. [file 1750-1326-3-15-S1.pdf]

1H NMR (500 MHz, DMSO-d_6) δ ppm 11.51 (br. s., 1 H) 8.57 (d, *J*=1.58 Hz, 1 H) 8.24 (dd, *J*=8.20, 1.89 Hz, 1 H) 7.76 (d, *J*=8.20 Hz, 1 H) 7.36 (d, *J*=8.20 Hz, 1 H) 7.19 (s, 1 H) 6.90 - 7.00 (m, 1 H) 4.48 (d, *J*=12.30 Hz, 1 H) 4.39 (t, *J*=7.09 Hz, 2 H) 4.21 (dd, *J*=14.03, 7.72 Hz, 1 H) 3.68 (d, *J*=11.03 Hz, 1 H) 3.35 - 3.45 (m, 1 H) 3.13 (td, *J*=13.79, 6.78 Hz, 2 H) 3.03 (m, 2 H) 2.91 (d, *J*=4.41 Hz, 3 H) 2.68 (s, 3 H) 2.36 (s, 3 H)

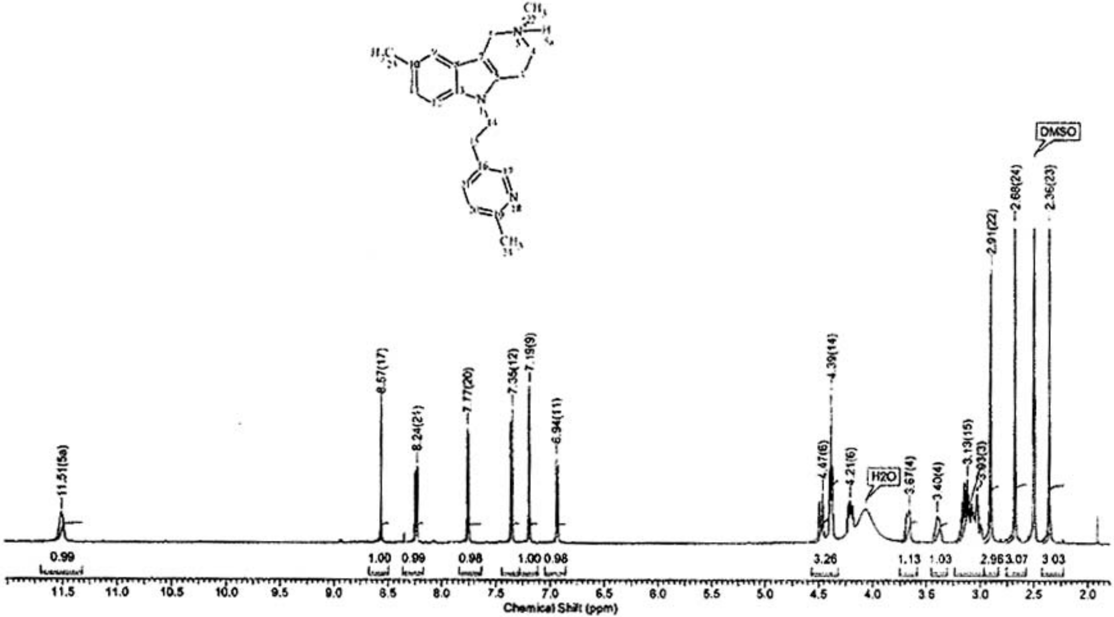

Figure 1. Proton spectrum in DMSO
