# Supplementary figures and images for: Evaluation of Dimebon in cellular model of Huntington's disease
Source: Mol Neurodegener. 2008 Oct 21;3:15. doi: 10.1186/1750-1326-3-15 (PMC2577671; doi:10.1186/1750-1326-3-15)

$^{13}\text{C}$  NMR (126 MHz,  $\text{DMSO}-d_6$ )  $\delta$  ppm 151.43 ; 146.46 ; 140.33 ; 135.37 ; 134.54 ; 131.02 ; 128.01 ; 127.33 ; 124.48 ; 122.96 ; 117.37 ; 109.76 ; 101.59 ; 50.11 ; 49.53 ; 43.01 ; 41.64 ; 31.74 ; 21.11 ; 19.32 ; 18.62 ;

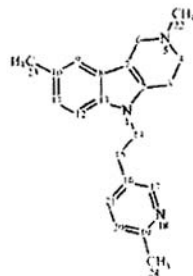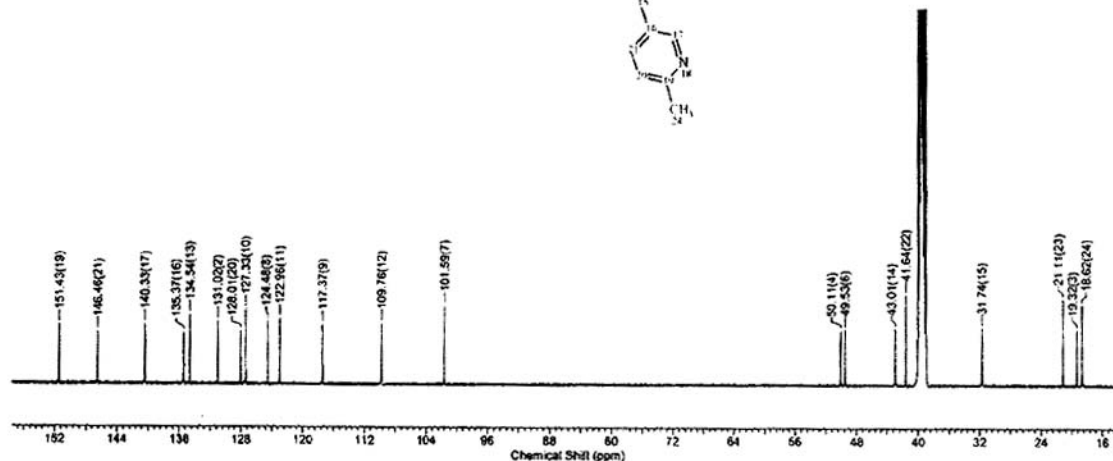

**Figure 2. Carbon spectrum in DMSO**

Supplement: Additional file 2 — The carbon 1D NMR spectra of Dimebon sample used in our experiments. The carbon 1D NMR spectra of Dimebon sample is shown. [file 1750-1326-3-15-S2.pdf]
