# Supplementary material for: Evaluation of Dimebon in cellular model of Huntington's disease
Source: Mol Neurodegener. 2008 Oct 21;3:15. doi: 10.1186/1750-1326-3-15 (PMC2577671; doi:10.1186/1750-1326-3-15)
Supplement: Additional file 3 — The results of Dimebon screening against a selected set of biochemical targets. The complete results obtained by MDS Pharma with a selected list of biochemical targets using 10 μM of Dimebon as a probe. The Cat numbers refer to the MDS Pharma Services assay specification. The receptors and species are listed. Dimebon was tested at 10 μM concentratio in duplicate. The % inhibition by Dimebon is shown numerically and graphically for each receptor. Significant targets (defined as % inhibition > 50%) are highlighted in yellow. [file 1750-1326-3-15-S3.pdf]

# EXPERIMENTAL RESULTS - BIOCHEMICAL ASSAYS

| | Cat. # | TARGET | BATCH* | SPP. | n= | CONC. | †% INHIBITION % | $IC_{50}$ | $K_I$ | $n_H$ | R |
|---|---|---|---|---|---|---|---|---|---|---|---|
| | 118050 | CYP450, 1A2 | 232511 | hum | 2 | 10 µM | 5 | | | | |
| ♦ | 118070 | CYP450, 2C19 | 232613 | hum | 2 | 10 µM | 55 | | | | |
| | 118060 | CYP450, 2C9 | 232612 | hum | 2 | 10 µM | 2 | | | | |
| | 118080 | CYP450, 2D6 | 232614 | hum | 2 | 10 µM | 16 | | | | |
| | 118090 | CYP450, 3A4 | 232615 | hum | 2 | 10 µM | 22 | | | | |
| | 140010 | Monoamine Oxidase MAO-A | 232466 | hum | 2 | 10 µM | 6 | | | | |
| | 140120 | Monoamine Oxidase MAO-B | 232467 | hum | 2 | 10 µM | 5 | | | | |
| | 164700 | Peptidase, BACE1 (β-Secretase) | 232778 | hum | 2 | 10 µM | -9 | | | | |
| | 200510 | Adenosine $A_1$ | 232649 | hum | 2 | 10 µM | 0 | | | | |
| | 200610 | Adenosine $A_{2A}$ | 232650 | hum | 2 | 10 µM | -3 | | | | |
| | 200720 | Adenosine $A_3$ | 232555 | hum | 2 | 10 µM | 6 | | | | |
| ♦ | 203100 | Adrenergic $\alpha_{1A}$ | 232528 | rat | 2 | 10 µM | 96 | | | | |
| ♦ | 203200 | Adrenergic $\alpha_{1B}$ | 232529 | rat | 2 | 10 µM | 100 | | | | |
| ♦ | 203400 | Adrenergic $\alpha_{1D}$ | 232530 | hum | 2 | 10 µM | 95 | | | | |
| ♦ | 203620 | Adrenergic $\alpha_{2A}$ | 232534 | hum | 2 | 10 µM | 97 | | | | |
| | 204010 | Adrenergic $\beta_1$ | 232625 | hum | 2 | 10 µM | -7 | | | | |
| | 204110 | Adrenergic $\beta_2$ | 232626 | hum | 2 | 10 µM | 14 | | | | |
| | 212510 | Bradykinin $B_1$ | 232432 | hum | 2 | 10 µM | -9 | | | | |
| | 212610 | Bradykinin $B_2$ | 232430 | hum | 2 | 10 µM | -2 | | | | |
| ♦ | 214510 | Calcium Channel L-Type, Benzothiazepine | 232556 | rat | 2 | 10 µM | 52 | | | | |
| | 214600 | Calcium Channel L-Type, Dihydropyridine | 232557 | rat | 2 | 10 µM | -12 | | | | |
| | 216000 | Calcium Channel N-Type | 232558 | rat | 2 | 10 µM | 0 | | | | |
| ♦ | 219500 | Dopamine $D_1$ | 232544 | hum | 2 | 10 µM | 70 | | | | |
| ♦ | 219700 | Dopamine $D_{2S}$ | 232546 | hum | 2 | 10 µM | 83 | | | | |
| ♦ | 219800 | Dopamine $D_3$ | 232547 | hum | 2 | 10 µM | 73 | | | | |
| ♦ | 219900 | Dopamine $D_{4.2}$ | 232548 | hum | 2 | 10 µM | 52 | | | | |
| | 225510 | Epidermal Growth Factor (EGF) | 232449 | hum | 2 | 10 µM | 1 | | | | |
| | 226300 | G Protein-Coupled Receptor GPR103 | 232731 | hum | 2 | 10 µM | 4 | | | | |
| | 226600 | $GABA_A$, Flunitrazepam, Central | 232692 | rat | 2 | 10 µM | 18 | | | | |
| | 226500 | $GABA_A$, Muscimol, Central | 232691 | rat | 2 | 10 µM | 6 | | | | |
| | 228610 | $GABA_{B1A}$ | 232480 | hum | 2 | 10 µM | -10 | | | | |

* Batch: Represents compounds tested concurrently in the same assay(s). ‡ Partially soluble in *in vitro* test solvent.
♦ Denotes item meeting criteria for significance
† Results with ≥ 50% stimulation or inhibition are highlighted.
R=Additional Comments
gp=guinea pig; ham=hamster; hum=human

# EXPERIMENTAL RESULTS - BIOCHEMICAL ASSAYS

| | Cat. # | TARGET | BATCH* | SPP. | n= | CONC. | †% INHIBITION (%) | $IC_{50}$ | $K_I$ | $n_H$ | R |
|---|---|---|---|---|---|---|---|---|---|---|---|
| | 232020 | Glucocorticoid | 232623 | hum | 2 | 10 µM | 3 | | | | |
| | 232700 | Glutamate, Kainate | 232436 | rat | 2 | 10 µM | 7 | | | | |
| | 232810 | Glutamate, NMDA, Agonism | 232437 | rat | 2 | 10 µM | 10 | | | | |
| | 232910 | Glutamate, NMDA, Glycine | 232656 | rat | 2 | 10 µM | 11 | | | | |
| | 233000 | Glutamate, NMDA, Phencyclidine | 232657 | rat | 2 | 10 µM | -5 | | | | |
| ♦ | 239610 | Histamine $H_1$ | 232563 | hum | 2 | 10 µM | 101 | | | | |
| ♦ | 239710 | Histamine $H_2$ | 232599 | hum | 2 | 10 µM | 96 | | | | |
| | 239810 | Histamine $H_3$ | 232701 | hum | 2 | 10 µM | -4 | | | | |
| ♦ | 241000 | Imidazoline $I_2$, Central | 232443 | rat | 2 | 10 µM | 89 | | | | |
| | 251600 | Melatonin $MT_1$ | 232495 | hum | 2 | 10 µM | 3 | | | | |
| | 252610 | Muscarinic $M_1$ | 232550 | hum | 2 | 10 µM | 9 | | | | |
| | 252710 | Muscarinic $M_2$ | 232551 | hum | 2 | 10 µM | 8 | | | | |
| | 252810 | Muscarinic $M_3$ | 232552 | hum | 2 | 10 µM | 7 | | | | |
| | 257010 | Neuropeptide Y $Y_1$ | 233075 | hum | 2 | 10 µM | 4 | | | | |
| | 257110 | Neuropeptide Y $Y_2$ | 232773 | hum | 2 | 10 µM | -5 | | | | |
| | 258590 | Nicotinic Acetylcholine | 232702 | hum | 2 | 10 µM | 3 | | | | |
| | 258700 | Nicotinic Acetylcholine α1, Bungarotoxin | 232444 | hum | 2 | 10 µM | 1 | | | | |
| | 260110 | Opiate δ (OP1, DOP) | 232688 | hum | 2 | 10 µM | 0 | | | | |
| | 260210 | Opiate κ (OP2, KOP) | 232559 | hum | 2 | 10 µM | 16 | | | | |
| | 260410 | Opiate µ (OP3, MOP) | 232560 | hum | 2 | 10 µM | 10 | | | | |
| | 264500 | Phorbol Ester | 232774 | mouse | 2 | 10 µM | 13 | | | | |
| | 265600 | Potassium Channel [$K_{ATP}$] | 232732 | ham | 2 | 10 µM | -3 | | | | |
| | 265900 | Potassium Channel hERG | 232455 | hum | 2 | 10 µM | 45 | | | | |
| | 268410 | Prostanoid $EP_4$ | 232629 | hum | 2 | 10 µM | 1 | | | | |
| | 268700 | Purinergic $P_{2X}$ | 232453 | rabbit | 2 | 10 µM | 5 | | | | |
| | 268810 | Purinergic $P_{2Y}$ | 232454 | rat | 2 | 10 µM | 6 | | | | |
| | 270000 | Rolipram | 232776 | rat | 2 | 10 µM | 3 | | | | |
| ♦ | 271000 | Serotonin (5-Hydroxytryptamine) $5\text{-}HT_1$, Non-Selective | 232636 | rat | 2 | 10 µM | 63 | | | | |
| | 271110 | Serotonin (5-Hydroxytryptamine) $5\text{-}HT_{1A}$ | 232637 | hum | 2 | 10 µM | 22 | | | | |

* Batch: Represents compounds tested concurrently in the same assay(s). ‡ Partially soluble in *in vitro* test solvent.
♦ Denotes item meeting criteria for significance
† Results with ≥ 50% stimulation or inhibition are highlighted.
R=Additional Comments
gp=guinea pig; ham=hamster; hum=human

# EXPERIMENTAL RESULTS - BIOCHEMICAL ASSAYS

| Cat. # | TARGET | BATCH* | SPP. | n= | CONC. | † % INHIBITION (%) | $IC_{50}$ | $K_I$ | $n_H$ | R |
|---|---|---|---|---|---|---|---|---|---|---|
| 271200 | Serotonin (5-Hydroxytryptamine) $5-HT_{1B}$ | 232638 | rat | 2 | 10 μM | 48 | | | | |
| ♦ 271600 | Serotonin (5-Hydroxytryptamine) $5-HT_2$, Non-Selective | 232639 | rat | 2 | 10 μM | 83 | | | | |
| ♦ 271700 | Serotonin (5-Hydroxytryptamine) $5-HT_{2B}$ | 232641 | hum | 2 | 10 μM | 74 | | | | |
| ♦ 271800 | Serotonin (5-Hydroxytryptamine) $5-HT_{2C}$ | 232642 | hum | 2 | 10 μM | 95 | | | | |
| 271910 | Serotonin (5-Hydroxytryptamine) $5-HT_3$ | 232643 | hum | 2 | 10 μM | -7 | | | | |
| 272000 | Serotonin (5-Hydroxytryptamine) $5-HT_4$ | 232644 | gp | 2 | 10 μM | 17 | | | | |
| ♦ 272100 | Serotonin (5-Hydroxytryptamine) $5-HT_{5A}$ | 232645 | hum | 2 | 10 μM | 99 | | | | |
| ♦ 272200 | Serotonin (5-Hydroxytryptamine) $5-HT_6$ | 232646 | hum | 2 | 10 μM | 101 | | | | |
| 278110 | Sigma $\sigma_1$ | 232456 | hum | 2 | 10 μM | 28 | | | | |
| 278200 | Sigma $\sigma_2$ | 232457 | rat | 2 | 10 μM | 22 | | | | |
| 279510 | Sodium Channel, Site 2 | 232447 | rat | 2 | 10 μM | 37 | | | | |
| 255510 | Tachykinin $NK_1$ | 232458 | hum | 2 | 10 μM | 20 | | | | |
| 220320 | Transporter, Dopamine (DAT) | 232532 | hum | 2 | 10 μM | 13 | | | | |
| 226400 | Transporter, GABA | 232433 | rat | 2 | 10 μM | -26 | | | | |
| 204410 | Transporter, Norepinephrine (NET) | 232531 | hum | 2 | 10 μM | 20 | | | | |
| 274030 | Transporter, Serotonin (5-Hydroxytryptamine) (SERT) | 232533 | hum | 2 | 10 μM | -7 | | | | |

* Batch: Represents compounds tested concurrently in the same assay(s). ‡ Partially soluble in *in vitro* test solvent.
♦ Denotes item meeting criteria for significance
† Results with ≥ 50% stimulation or inhibition are highlighted.
R=Additional Comments
gp=guinea pig; ham=hamster; hum=human
